# MRI Safety Considerations for Permanent Magnet Implants in Muscle

**DOI:** 10.1002/jmri.70126

**Published:** 2025-09-17

**Authors:** Cameron R. Taylor, Eric D. Anttila, Steven J. Charlebois, David C. Gross, Amanda F. Taylor, Jose O. Negron‐Garcia, Christopher E. Suckow, L. Tiffany Lyle, Scott R. Hooten, Seong Ho Yeon, Christopher C. Shallal, Hugh M. Herr

**Affiliations:** ^1^ K. Lisa Yang Center for Bionics Massachusetts Institute of Technology Cambridge Massachusetts USA; ^2^ Lampe Joint Department of Biomedical Engineering UNC‐Chapel Hill and NC State Chapel Hill North Carolina USA; ^3^ MED Institute West Lafayette Indiana USA; ^4^ Cook Research Incorporated West Lafayette Indiana USA

**Keywords:** intramuscular implants, magnetic implants, MRI conditionality, permanent magnet implants

## Abstract

**Background:**

Permanent magnet implants are used with several medical and assistive devices, such as cochlear implants, dental attachments, and prosthetic control, but raise caution for MR imaging. Previous work has evaluated several magnet implants for position and magnetization stability, as well as for image artifacts under MRI. Yet, the intramuscular magnets used for prosthetic control still require evaluation for potential MRI conditionality.

**Purpose:**

To investigate the position and magnetization stability of and image artifacts from 3‐mm‐diameter spherical permanent magnets (*B*_r_ = 1.393 T, *H*_ci_ = 1.637 MA/m) implanted within muscle.

**Study Type:**

Prospective longitudinal study.

**Animal Model:**

Porcine; one animal, eight muscles.

**Field Strength/Sequence:**

0.55‐T, 1.5‐T/SE, GRE.

**Assessment:**

Permanent magnets and nonmagnetic controls were implanted into eight muscles and exposed to 1.5‐T MRI 36 days post‐implantation. All sites were examined histologically for evidence of implant migration (acute fibrotic response or fibrotic capsule disruption). Benchtop studies evaluated worst‐case demagnetization and image artifacts (artifact radius minus implant radius). The primary measure of position stability was histological examination interpreting characteristics of progressive skeletal muscle healing. Secondary position stability analysis was performed via CT imaging.

**Statistical Tests:**

Unpaired one‐sided sign test with a significance level of 0.05. Demagnetization and imaging artifacts were summarized as maximums.

**Results:**

Fibrotic capsules were similarly intact at permanent magnet and control sites (fibrotic capsule thicknesses: 20–550 μm [magnets], 20–220 μm [controls]). No effect of MRI exposure on implant migration was observed via secondary analysis (*p* = 0.965 [0.55‐T], *p* = 0.996 [1.5‐T]). Maximum demagnetization was 2.1% under 0.55‐T exposure and 13.5% under 1.5‐T exposure, and maximum image artifact was 71 mm at both imaging strengths.

**Data Conclusion:**

The permanent magnet implants used in this study were resistant to migration and substantial demagnetization under 0.55‐T and 1.5‐T MRI exposure and resulted in negligible image artifacts for critical organ imaging, suggesting that the presence of these implants does not preclude a patient from receiving MR imaging up to 1.5T.

**Evidence Level:**

N/A.

**Technical Efficacy:**

Stage 5: Improvements in patient care.

## Plain Language Summary

Some people have magnets implanted in their bodies. Implanted magnets let people control prostheses, let people attach things to themselves, and let doctors control things inside the body. But can a person be imaged by MRI if they have a magnetic implants? The authors checked to see if sesame‐seed‐sized neodymium magnets in muscle could tolerate a 1.5‐T MRI, and they found that the magnets remained magnetized and did not move. These results mean that people with implanted magnets can still benefit from up to 1.5‐T MRI.

## 1 Introduction

Permanent magnet implants provide a variety of advantages in the contexts of medical, aesthetic, and assistive devices. They facilitate attachment and alignment, as in cochlear implants [1], bone conduction implants [2] and breast tissue expander magnetic infusion ports [3], as well as attachment and retention, as in snap‐on dentures [4] and aesthetic prostheses [5]. Permanent magnets are also used to enable actuation of implanted medical devices, such as magnetically controlled growth rods [6] and blood pumps [7]. The use of magnetic implants in various surgical procedures has also grown steadily over the past 50 years [8]. Recently, surgeons working together with engineers have also implanted permanent magnets into muscles to enable real‐time control over prostheses [9].

**FIGURE 1:**
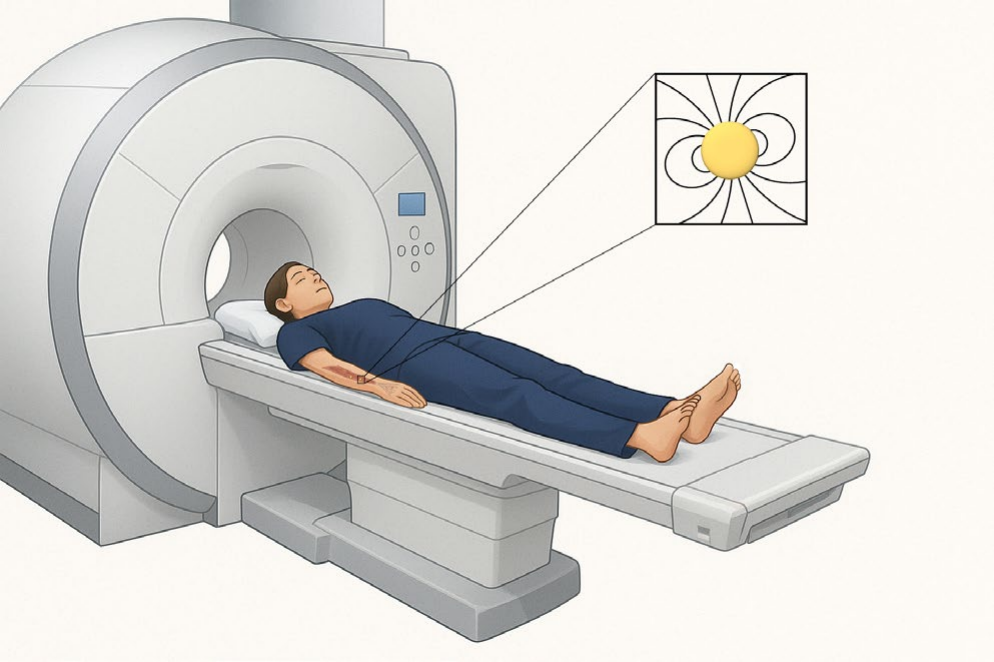
MR Imaging of a patient with permanent magnets implanted in a muscle. In the case of muscle interfaces such as magnetomicrometry, small permanent magnets are implanted into a patient's muscles for control over a prosthesis or other device. The effects of these magnets on the MRI image, and the effects of the MRI on the magnets, are considered in this work.

While magnetic implants provide various medical benefits, they also raise caution for MR imaging. MR imaging provides substantial clinical benefits, allowing many diagnostics to be performed non‐invasively. The above devices have various levels of MRI conditionality, even varying from one product to another within a given application. In light of recent findings on the societal cancer risk burden of CT scans [10], the proportion of diagnostics performed via MRI will likely continue to increase as MR imaging methods expand in diagnostic usefulness to a larger span of tissues and pathologies [11], as MRI machines become more accessible [12], and as MR imaging speed increases [13]. Thus, it is important that the effects of MRI on magnetic implants, as well as the effects of the implants on the MR images, be well understood. Specifically, permanent magnet implants should be evaluated for their (i) position stability against migration in tissue, (ii) resistance to demagnetization, and (iii) extent of imaging artifacts. In this work, we aimed to provide a framework to evaluate a permanent magnet implant in these ways to test for potential MRI conditionality, within the specific context of 1.5‐T imaging with small permanent magnets implanted within a muscle (see Figure 1).

## 2 Materials and Methods

This work was approved by the Institutional Animal Care and Use Committees at MED Institute, Purdue University, and the Massachusetts Institute of Technology. One animal (one female domestic swine, 54 kg) was procured from Oak Hill Genetics (Ewing, IL) and cared for in the institution's animal care facility in accordance with institutional guidelines. The animal study and histological analysis were performed under good laboratory practice (GLP) compliance (USFDA, Code of Federal Regulations, Title 21, Part 58—Good Laboratory Practice for Nonclinical Laboratory Studies).

The permanent magnet implants tested in this work are described in more detail in previous work [14]. Briefly, the implants are 3‐mm‐diameter spherical permanent magnets (3 mm + 0.020/−0.070 mm) that have been coated with approximately 5 μm of gold and 21 μm of Parylene C. The magnets are grade N48SH, with manufacturer‐reported parameters of residual flux density *B*_r_ = 1.393 T and intrinsic coercivity *H*_ci_ = 1.637 MA/m. Before beginning testing, we exposed the magnets to 3‐T magnetic fields both in simulation and empirically, and we used these first results to narrow the scope of the experiments to 1.5‐T exposure and less.

We performed force and torque testing according to ASTM Standards F2052‐21 [15] and F2213‐17 [16] (see Supporting Information: Section 1 as well as Figures S1 and S2 for details of this testing). Measurements were performed in 0.55‐T and 1.5‐T scanners with measurements of the magnetic fields and gradients, and then results were converted into values expected at worst‐case conditions. However, though position stability is often considered in the context of standard force and torque measurement testing [17], noting that dislodgement is tissue‐attachment specific [18], these force and torque measurements were measured solely for reference, with no intention to use them as a primary determinant of implant position stability.

**FIGURE 2:**
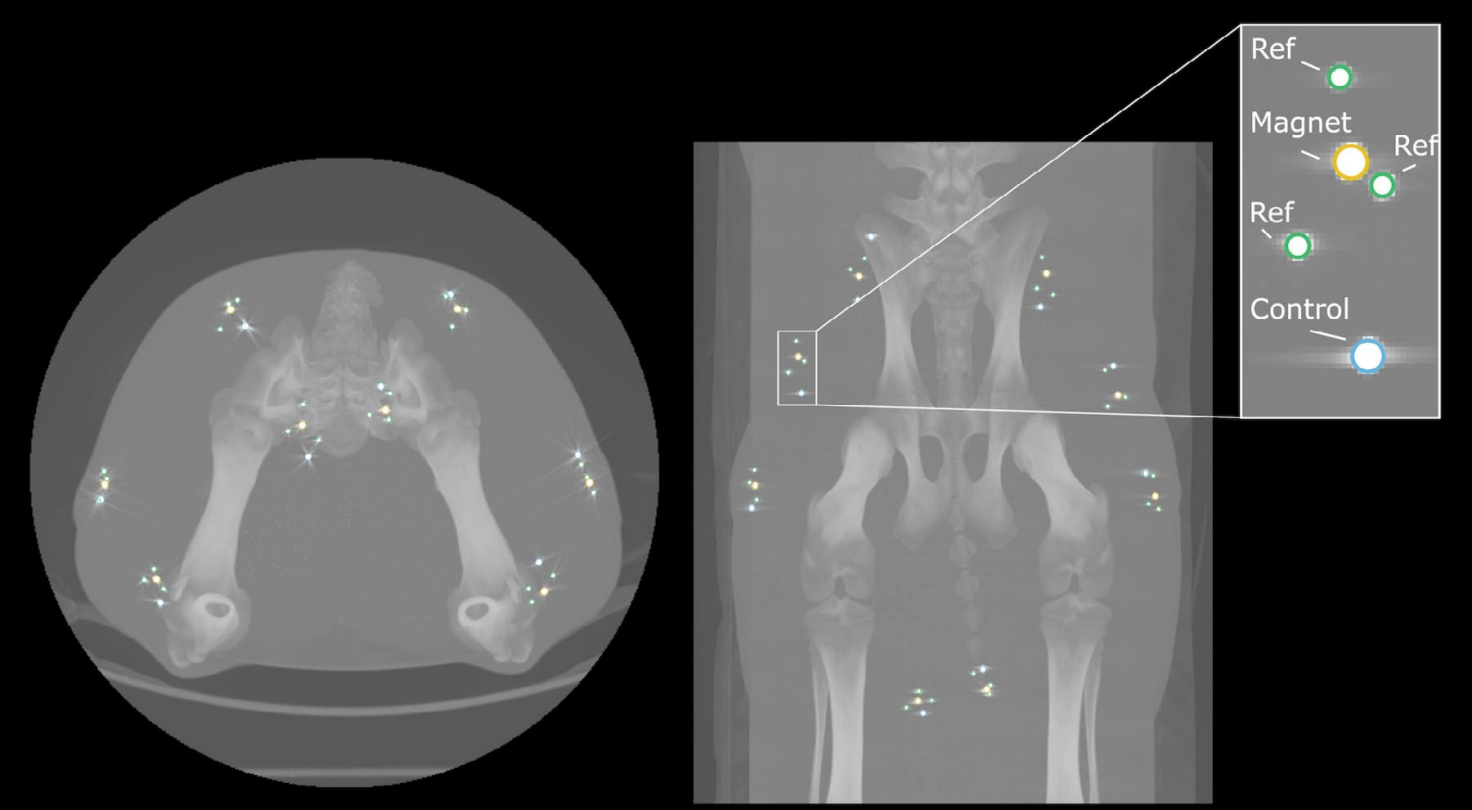
Analysis of permanent magnet and nonmagnetic control implant positions pre versus post MRI exposure. In each of eight muscles in one pig, we implanted a permanent magnet (highlighted in gold) and a nonmagnetic (tantalum) control (highlighted in blue). The main strategy we used to evaluate potential migration was histology. However, as a secondary evaluation of implant position stability, we also implanted three nonmagnetic (tantalum) position reference implants (highlighted in green) around each magnet. These reference implants provided a reference coordinate system to evaluate differences in position measurements within the computed tomography (CT) images for the magnets and controls. The left and right images represent transverse and dorsal views, respectively. The implants were placed in the left gluteus medius (GM), biceps femoris (BF), rectus femoris (RF), and semimembranosus (SM) muscles, and into the right SM, RF, BF, and GM muscles (listed here corresponding roughly to their clockwise arrangement in the left image).

To test for position stability, we implanted one permanent magnet (described above), one nonmagnetic control (2.73‐mm‐diameter tantalum sphere, B2730‐BU2‐S00, X‐medics, Hvidovre, Denmark), and three reference implants (1.6‐mm‐diameter tantalum spheres, B1600‐BU2‐S80, X‐medics) into each of eight muscles in one pig. The nonmagnetic control was used both as a histological control and as a CT perceived movement control, and the reference implants were used as position references to calculate the perceived movement of the magnet and control before versus after MRI exposure. Figure 2 details the locations of these implants. Implantation was performed similarly to methods described in other work [14]. For a representative photograph of a test site, see Figure S3. All control implants were cleaned and passivated according to ASTM F86 [19] and autoclaved before implantation. Control implants were coated with identical methods to the permanent magnet implants, with 20–30 μm Parylene C applied in a class 10,000 cleanroom (Specialty Coating Systems, Clear Lake, Wisconsin, USA). The animal was exposed to 1.5‐T MR imaging (31 min) at 36 days post‐implantation (following an earlier exposure to 0.55‐T MR imaging at 28 days post‐implantation) and was kept alive for 72 h. The animal was then euthanized, and the magnet and control implant sites were harvested and fixed in 10% neutral buffered formalin for histological examination.

To determine each implant location, we iteratively repositioned a small titanium wire ring on the top of each sample between consecutive high‐resolution radiography images (XPERT 80‐L Digital Radiography Machine, KUBTEC Scientific, Stratford, Connecticut, USA) and marked the implant location with tissue dye (MER DYE8R, Mercedes Scientific, Lakewood Ranch, Florida, USA) once the implant was centered within the ring, as seen in the image. We then used a nonmagnetic (ceramic) knife to trim the samples down to 1 cm cubes centered around each implant. We progressively dehydrated the samples with reagent alcohol (ACS grade, anhydrous; ~90% ethanol, ~5% methanol, ~5% isopropanol; RS4029; Avantik, Pine Brook, New Jersey, USA) for 4 h at each of 70%, 95%, and 100%, cleared the tissues with xylene, and embedded each in Spurr resin. Again using high‐resolution radiography, and with a straight titanium wire, we marked the precise location of each implant within the Spurr resin blocks. We sawed through each block directly through the center of the implant using a saw microtome equipped with a diamond blade (SP1600, Leica Biosystems, Nussloch, Germany), then used a drill bit and drill press to eject the remaining portion of the implant from behind. We then sectioned each block at 5‐μm thickness on a rotary microtome (HistoCore Autocut, Leica Biosystems) and stained all slides with hematoxylin and eosin. LTL, a board‐certified veterinary pathologist with 13 years of experience, then interpreted characteristics of progressive healing associated with magnet and control implants within the target tissues, including any alterations to the skeletal muscle, proliferation of fibrovascular tissue, inflammation, necrosis, or other pathologically relevant changes around the implants.

**FIGURE 3:**
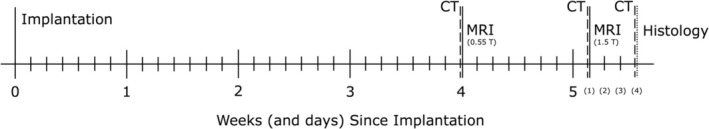
Timeline for computed tomography (CT) imaging, MRI exposure, and histological analysis. Dashed lines indicate CT scans, and the dotted line indicates the time point for histological examination.

For the secondary position stability analysis, we imaged via CT (GE LightSpeed VCT; helical mode, 120 kVp, 350 mA, 1 s rotation time, 0.625 mm slice thickness, 0.977 mm pixel spacing, 500 mm reconstruction diameter, “detail” convolution kernel, body filter) immediately before 0.55‐T MRI exposure (Siemens Magnetom Free.Max, 32 min), then again 8 days later, immediately before 1.5‐T MRI exposure (Signa, GE). We then imaged via CT one final time 3 days post‐exposure to 1.5‐T MRI, immediately before euthanasia. We waited 4 weeks before the initial (0.55‐T) MRI exposure (see Figure 3) to allow for fibrosis and healing around the implants post‐implantation. To enable quantitative analysis of the implant positions, we placed the position reference implants non‐collinearly around each permanent magnet implant, and we used these reference implants to transform the magnet and control positions from their post‐exposure coordinate system into their pre‐exposure coordinate system using an affine transformation, enabling us to calculate how much the transformed position measurement changed from one CT scan to another. We will hereafter refer to this change in these measured positions as the “perceived movement” of the magnet and control implants. For additional information on the CT scanning and image analysis, see Supporting Information: Section 2.

**FIGURE 4:**
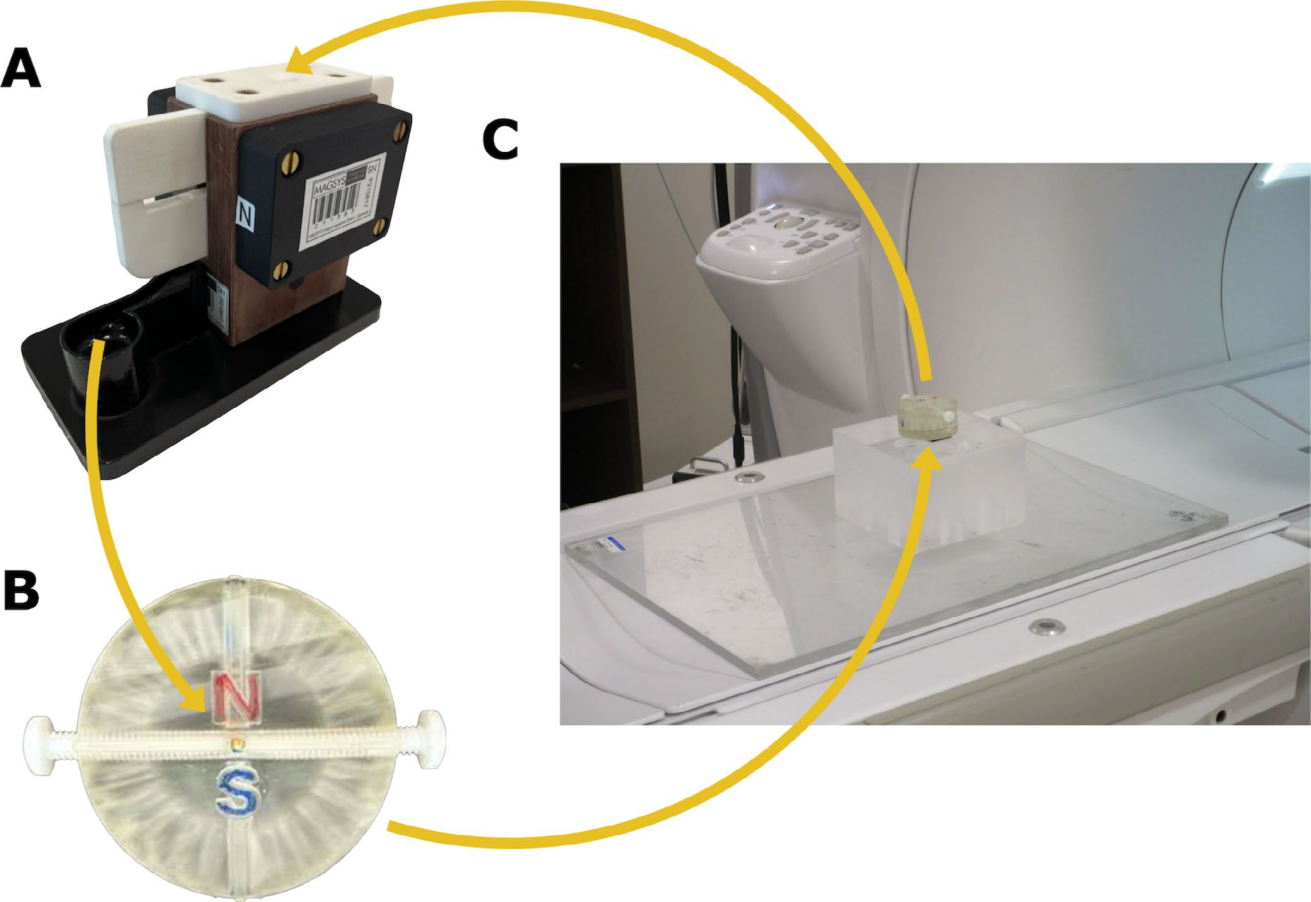
Magnetization strength of permanent magnets pre and post MRI exposure. (A) The magnetization strength (residual flux density) of each permanent magnet was measured using a custom Helmholtz coil measurement device. (B) Each magnet was then affixed at a predetermined angle in a custom fixture. (C) Each magnet was then exposed to MRI‐strength fields and gradients during imaging in the fixture at iso‐center (image shows fixture before movement into iso‐center), after which it was then measured again using the Helmholtz coil measurement device.

To test for resistance of the permanent magnet implants to demagnetization, we exposed 13 of the magnets to 0.55‐T imaging and another 13 to 1.5‐T imaging. Before exposure, we measured the strength of each magnet using a custom‐built Helmholtz coil with an alignment device (HHC20/SN211383, MAGSYS Magnet Systeme, Dortmund, Germany, see Figure 4A) and a fluxmeter (FG16, MAGSYS). The Helmholtz coil consists of two 4000‐turn coils (27 mm ID, 53.2 mm OD, 11 mm thickness), and the fluxmeter performs the measurement via the Helmholtz coil based on the principle of Faraday's law of induction [20]. To perform the measurement, we held the magnet far (at least 0.5 m) away, reset the fluxmeter, then placed the magnet in the center of the Helmholtz coil and recorded the magnetization strength. After measuring the strength of each magnet, we then placed it into a custom 3D‐printed test fixture (see Figure 4B) with ports for alignment to temporarily inserted bar magnets, and with nylon screws to affix the magnet orientation before the removal of the bar magnets. Noting that demagnetization is dependent not just on the strength, but also the angle of the exposed field [17], we aligned the magnet to one of 13 orientations (using tabs on the fixture in 15° increments) and placed the fixture within the MRI at isocenter for approximately 1 min, thereby exposing it to the imaging field. After exposure, we measured the strength again using the Helmholtz coil device. To account for any possible drift of the measurement system, we measured two unexposed permanent magnets at the same time as the pre‐and post‐measurements as a measurement control and ensured no non‐negligible drift had occurred. For comparison, we also simulated all exposure strengths and angles for the given magnet material and geometry via COMSOL. Finally, noting that demagnetization can be dependent on the duration of magnetic field exposure [21], to account for any temporal effects of exposure duration, we then exposed 7 additional permanent magnets to 1.5‐T imaging, following the methods above with all at worst‐case orientation (opposite the exposed field) and for varying durations of 1, 2, 4, 8, 16, 32, and 64 min.

We tested for MRI imaging artifacts following ASTM F2119‐07 (2013) [22]. To test for MRI imaging artifacts, we performed both 0.55‐T (Siemens Free.Max) and 1.5‐T (Siemens Altea) imaging of a container of copper sulfate solution (2 g/L) with and without the presence of one of the permanent magnets. When present, the permanent magnet was submerged and suspended on a mesh net (see Figure S4). We imaged with both spin echo (TR = 500 ms; TE = 20 ms) and gradient echo (TR = 100 ms; TE = 15 ms [0.55‐T imaging] and 10 ms [1.5‐T imaging]) sequences in all imaging planes, with a slice thickness of 5 mm for all images at both strengths. To ensure accuracy, we used a plastic rod to verify each MRI system's pixel size. For all slices in all planes, we subtracted the reference image from the implant image and divided by the reference image to compute a percent change, then performed binary thresholding of all pixels at a 30% threshold, as |(*p*_implant_‐*p*_ref_)/*p*_ref_| > 0.3. For each plane and each imaging modality, we then computed the maximum image artifact extent as half the maximum vertical or horizontal span of the positive threshold minus the implant radius.

## 3 Statistical Analysis

For the secondary position stability analysis, we applied a one‐sided unpaired sign test for the median under the null hypothesis that the median of the magnet perceived movement would not be greater than the median of the control perceived movement (noting that, due to the reference implants being closer to the magnet, the controls would likely be more affected by any muscle hypertrophy or atrophy or changes in muscle tone, and thus should have a greater median if the MRI had no effect). Calculations were performed using the cumulative distribution function via Python SciPy 1.16.1 (Python Software Foundation, Wilmington, Delaware, USA). A value of *p* < 0.05 was considered statistically significant.

## 4 Results

**FIGURE 5:**
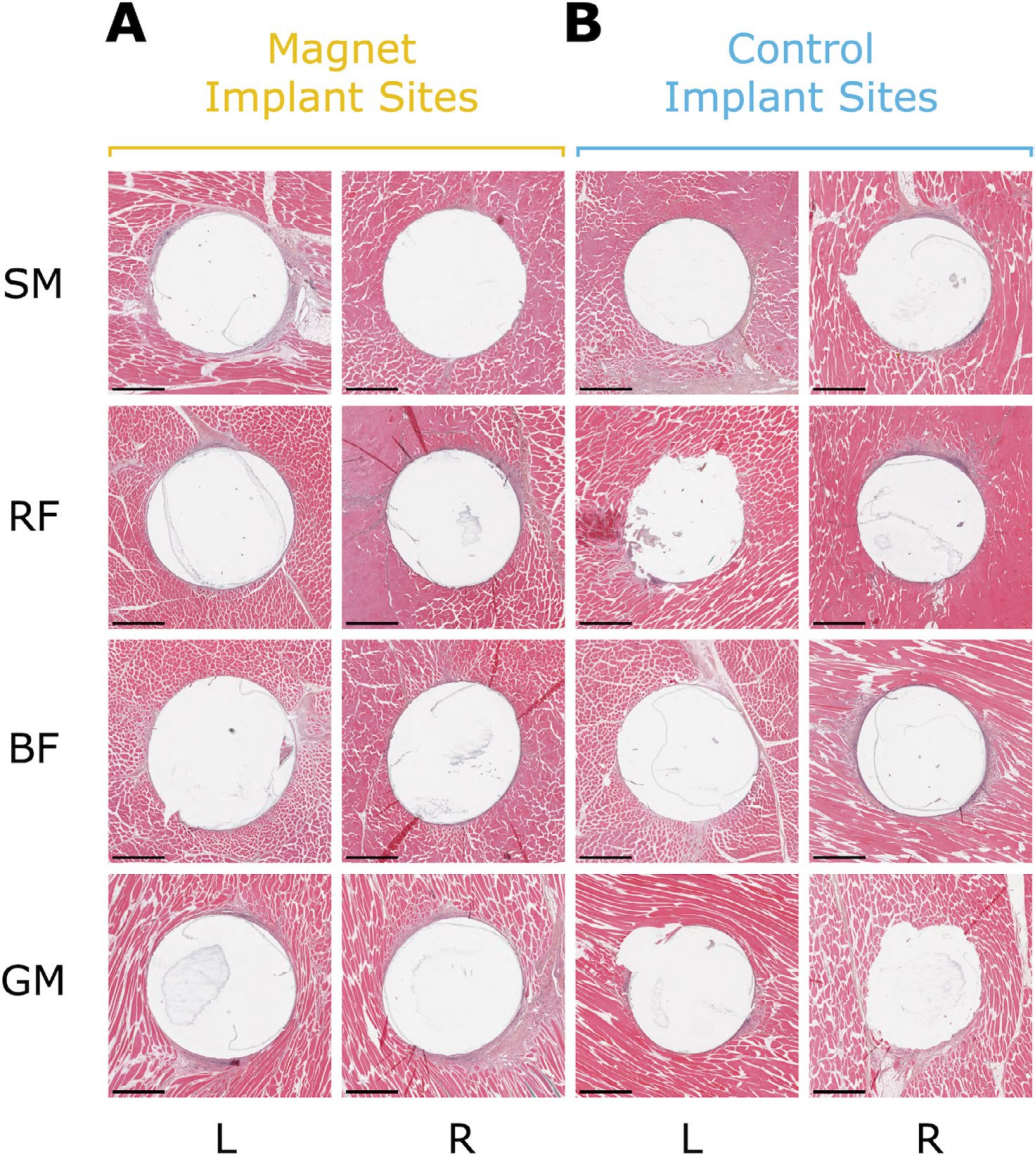
Histopathology Results. Spurr resin embedded cross sections of each implant site, sectioned at 5‐μm thickness and stained with hematoxylin and eosin (we sawed each sample in half through each implant after embedding, then ejected the implants before sectioning). The eight images on the left (panel A) are sections from the permanent magnet implant sites, and the eight images on the right (panel B) are sections from the nonmagnetic control implant sites. Each slide shows a 4.5 × 4.5 mm square of the implant cross section centered on the implant (a 1‐mm scale bar is provided in the bottom left hand corner of each slide). Rows correspond to muscles (semimembranosus, rectus femoris, biceps femoris, and gluteus medius) and columns correspond to the left versus right side of the animal. Note the fibrotic capsule around each implant site, and the remnants of the Parylene C coating, can be observed in many of the slides.

We did not observe any histologic evidence of migration (inflammation or necrosis) of any magnet or control implants during the duration of the study (see Figure 5). We observed a thin fibrous capsule surrounding each of the implant sites. This fibrous capsule measured between 20 and 550 μm in thickness at the permanent magnet implant sites and between 20 and 220 μm in thickness at the nonmagnetic implant sites. The biological response to both the magnetic implants and the control implants showed an appropriate biological response, with minimal tissue response and healing progressing as expected. For a more detailed histological analysis, see Supporting Information: Section 3.

**FIGURE 6:**
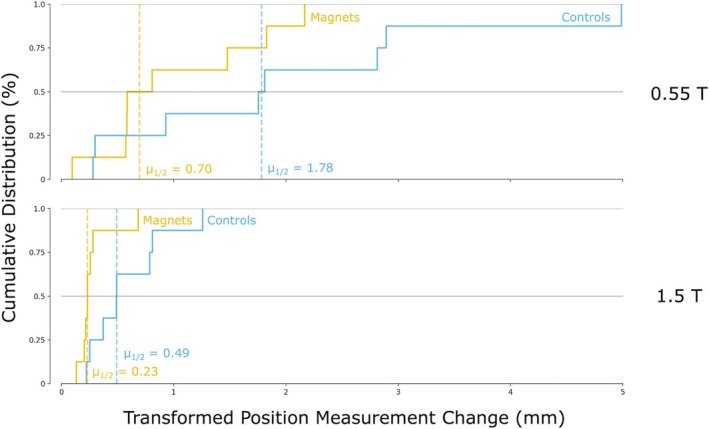
Measurement differences pre versus post MRI exposure. These cumulative distribution functions show the likelihood for the permanent magnet implants to have been measured with various perceived movements in comparison with the nonmagnetic control implants. The nonmagnetic control implants measurement changes were in general larger, likely due to the controls being located farther away from the reference implants, and thus more sensitive (through coordinate transformation) to changes in muscle morphology over time (the CT scans for the 0.55‐T exposure study were 8 days apart, while the CT scans for the 1.5‐T exposure study were only 3 days apart). For a table providing all data point values in these plots, see Table S1.

We also did not observe a significant MRI‐exposure effect in our secondary analysis via CT imaging (*p* = 0.965 for 0.55‐T and *p* = 0.996 for 1.5‐T). In the 0.55‐T exposure analysis, the medians were 0.70 mm and 1.78 mm for the magnet and control implants, respectively, with two of eight magnet measurement changes above the control median (see the top plot of Figure 6). In the 1.5‐T exposure analysis, the medians were 0.23 and 0.49 mm for the magnet and control implants, respectively, with one of eight magnet measurement changes above the control median (see the bottom plot of Figure 6). All magnet perceived movements were found to be small (maximums of 2.17 mm across the 0.55‐T exposure study and 0.68 mm across the 1.5‐T exposure study), in agreement with the histological examination that no migration of the implants occurred through the tissue.

We found force and torque values of 0.35 N and 4.19 mNm for the 0.55‐T exposure, respectively, and force and torque values of 0.94 N and 1.23 mNm for the 1.5‐T exposure, respectively (in both cases having converted the force values upward to account for a desired exposure to a gradient of 20 T/m, as discussed in Supporting Information: Section 1).

**FIGURE 7:**
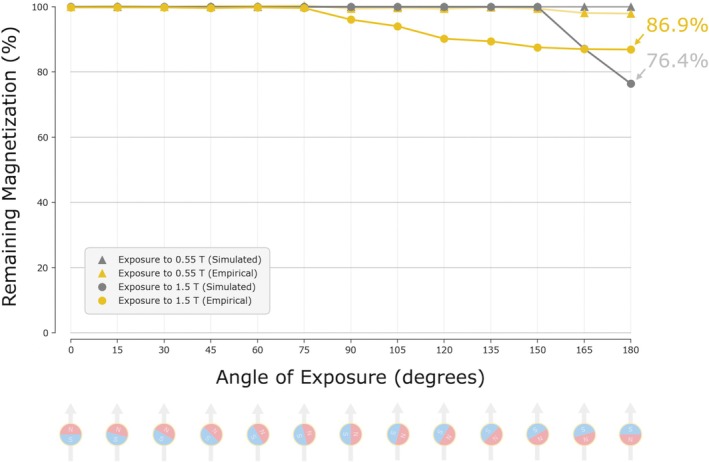
Demagnetization (empirical and simulated) magnetic spheres post exposure to 0.55‐T and 1.5‐T MRI. Remaining residual flux density post exposure to 0.55‐T and 1.5‐T MRI, with each magnet rigidly‐affixed at a given angle to the applied field.

When sweeping through the angles ranging from when aligned with the magnetic field (0 degrees) to directly opposing it (180 degrees) in 15‐degree increments (each exposure being performed with a different magnet), a maximum of 2.1% demagnetization was observed under 0.55‐T exposure, and a maximum of 13.5% demagnetization was observed under 1.5‐T exposure, with these maximums both occurring when the magnet was rigidly fixed in the orientation directly opposing the magnetic field of the MRI (see Figure 7).

**FIGURE 8:**
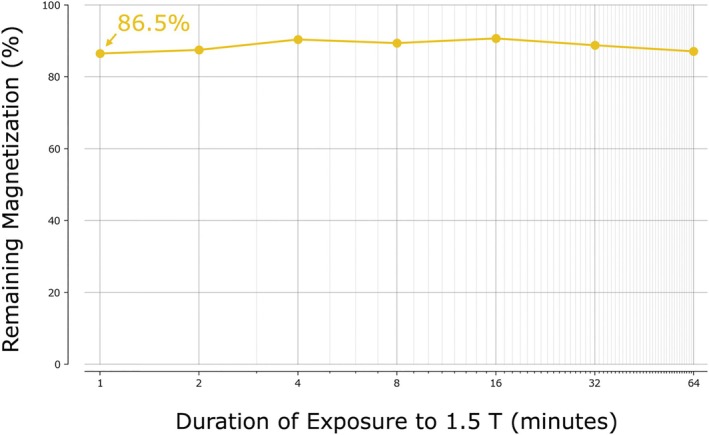
Demagnetization (empirical) magnetic spheres under worst‐case exposure to 1.5T MRI at varying durations. Remaining residual flux density post exposure to 1.5T MRI for various durations, with the magnet rigidly‐affixed in direct opposition to the applied field. The horizontal axis is shown in minutes and is spaced logarithmically for ease of viewing, while the vertical axis shows the percentage of magnetization remaining post exposure of one of the fully‐magnetized permanent magnets to the 1.5‐T MRI.

The duration of exposure to 1.5‐T imaging at the worst‐case orientation was not found to influence the amount of demagnetization (see Figure 8). The maximum demagnetization found was 13.5%, and occurred after the shortest duration.

**FIGURE 9:**
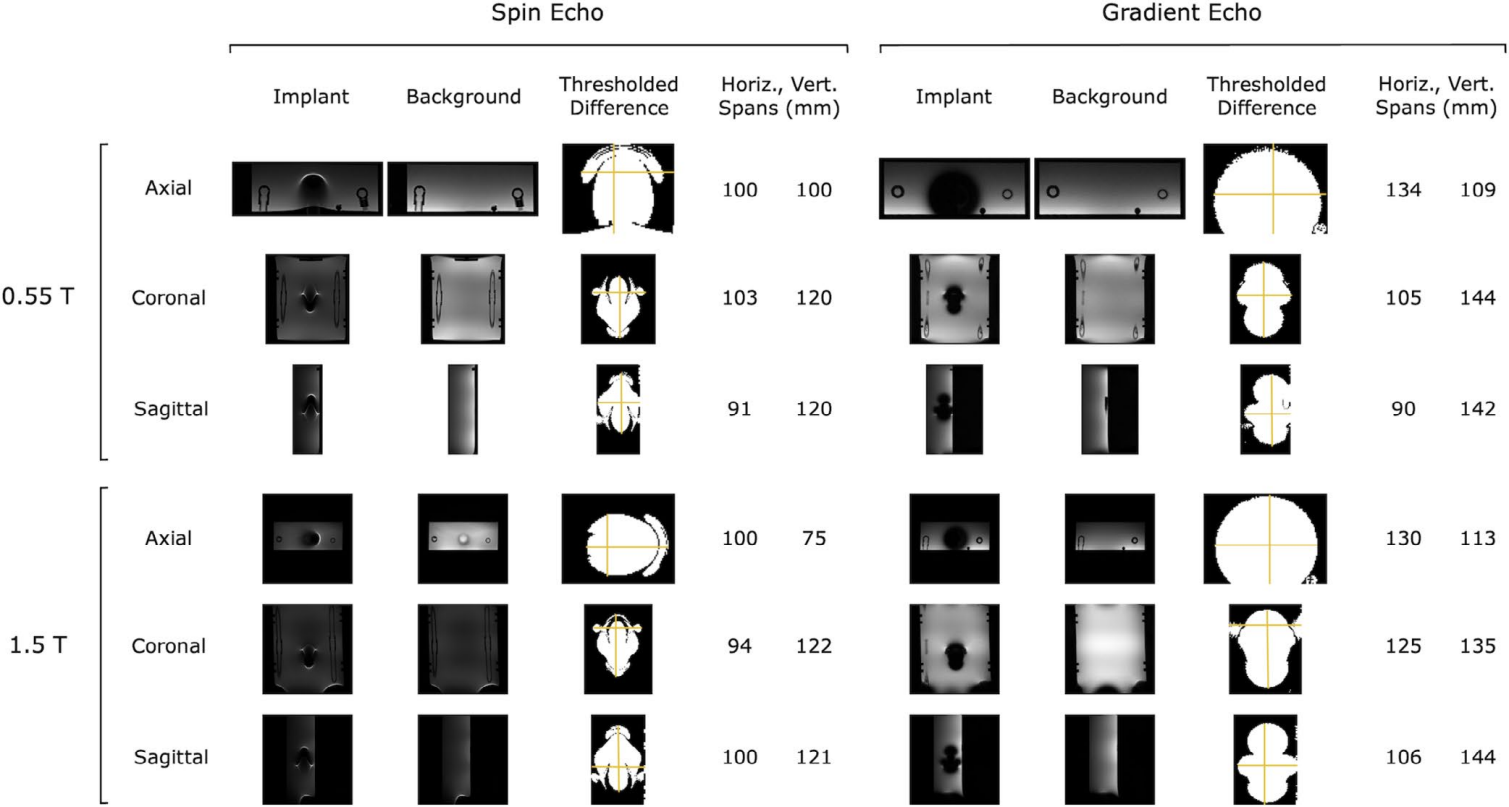
Image artifacts resulting during 0.55‐T and 1.5‐T imaging. Image artifacts in 0.55‐T and 1.5‐T MRI images from the permanent magnet implant are shown, with 0.55‐T images on top, 1.5‐T images on bottom, spin echo images on the left, and gradient echo images on the right. Original images of the implant and of the background are shown in each plane, along with the thresholded difference image and the span markings (gold lines). The horizontal and vertical spans are listed next to each thresholded difference image. Note that the spans are labeled here, and that the image artifact extent is calculated as half the span minus the implant radius. All images show thresholded differences, thresholded at 30% change from pre to post introduction of the implant. Note that the image for each plane was collected separately, and also that the images in the figure are not scaled relative to one another. The axial, coronal, and sagittal images are shown from top to bottom in each of the panels.

MR imaging artifacts of 59 and 71 mm were observed for spin echo and gradient echo sequences, respectively, under 0.55‐T imaging, and imaging artifacts of 60 and 71 mm were observed for spin echo and gradient echo sequences, respectively, under 1.5‐T imaging. See Figure 9 for a visual of the image artifacts during 1.5‐T imaging and Figure S5 for a visual of the image artifacts during 0.55‐T imaging.

## 5 Discussion

The permanent magnet implants we investigated were resistant to migration under 1.5‐T MRI exposure 5 weeks post‐implantation, maintained most (86.5%) of their magnetization strength under repeated 1.5‐T MRI exposure, and exhibited image artifacts that were not unsubstantial, and yet which do not preclude the diagnostic scanning of critical organs.

Based on the results observed, it is unlikely that these permanent magnets migrate in muscle under up to 1.5‐T MRI exposure after 5 weeks post implantation. There was no histologic evidence of migration of magnet or control implants in the examined sections, with all implant sites displaying appropriate biocompatibility with normal histological tissue response. The 20‐ to 550‐μm‐thick fibrotic capsules observed surrounding the permanent magnet implants may partially explain the position stability of the magnetic implants against migration under 1.5‐T MRI exposure. Advanced healing was progressing as expected in the skeletal muscles implanted with the magnet and control implants, and it is anticipated that this healing would have continued without complication had the study endpoint been extended. Our CT imaging observations do not disagree with these primary results from histological examination. We selected unalloyed tantalum (ASTM F560 [23]) for the control and reference implants because tantalum is nonmagnetic and thus should not be affected by exposure to magnetic fields. While histological examination was our primary measure of position stability, we also wished to view the implants via CT imaging pre and post exposure to 1.5‐T MR imaging, as well as pre and post exposure to lower‐strength 0.55‐T MR imaging. This way, if via histological examination, we observed that migration had occurred, the CT imaging analysis could aid in determining whether the migration occurred at 0.55‐T or 1.5‐T exposure. We note the importance of having a patient wait for 5 weeks post implantation for the results of this study to be relevant, as we explicitly included this time delay to allow for fibrosis and healing at the implant sites.

The amount of permissible demagnetization of a permanent magnet implant is context‐specific. In the context of tracking the distance between magnetic beads, the maximum amount of demagnetization we observed (remaining residual flux density of 86.5%) is insufficient to render these implants ineffective for tracking and prosthetic control; a 13.5% drop in magnetization strength is equivalent to only a 17% increase in noise level at 4.5 cm (see Supporting Information: Section 4), corresponding to an acceptable reduction in tracking ability. We also found the size of the drop in magnetization strength to be independent of the duration of the 1.5‐T MRI exposure.

The maximum size of the imaging artifacts that we observed was 71 mm for both 0.55‐T and 1.5‐T imaging, similar to what has been found in prior work imaging a permanent magnet [24]. These artifacts suggest that there would be no issue with a patient being able to obtain a clinically useful image of critical organs in parts of their body sufficiently far from the implant sites.

The heating of an implant due to MRI is another important consideration when considering MRI safety (see ASTM F2182‐19e2 [25]). Because the permanent magnet implants used in muscle tissue length tracking are less than 2 cm in diameter and are always at least 3 cm away from one another, the implants are expected to generate a negligible temperature increase during normal imaging [26].

## 6 Limitations

The results of this work only directly apply to Parylene‐C‐coated spherical N48SH magnets of the given dimensions and coercivity used in this study that are implanted in muscle. Different coatings or geometries may affect the biological response and tissue mechanical interface, and thus result in different position stability against migration. In addition, the geometry affects the demagnetization factor [27], and this factor combined with the coercivity of the implant determines its potential to be demagnetized. See Supporting Information: Section 5 for a discussion of the relationship of the exposed field, magnet material properties, and magnet geometry in the context of demagnetization.

The results of this study only apply up to 1.5T. One would need to repeat these studies at a higher field strength exposure for conclusions at higher MRI field strength exposures. As discussed in the Section 2 and in Supporting Information: Section 5, we ran a preliminary data collection exposing these magnets to 3T and found that under worst‐case conditions—when rigidly affixed at the worst possible angle—it is possible for the magnets to be fully demagnetized at that higher field strength. However, when a magnet is able to rotate, this mitigates its demagnetization in an MRI [28], and it is unknown how much ability to rotate is afforded by the flexibility of muscle tissue or how such a rotation may mitigate the demagnetization of these implants in a 3‐T MRI. Without further investigation, at the time of this paper, we do not recommend exposing intramuscular magnetic bead implants of these particular specifications to field strengths of 3‐T imaging or above.

The CT imaging analysis provided further insight into the positions and position stability of the permanent magnet and control implants, but it could be improved upon if desired to be used as a standalone method. The use of only three position reference implants limited us to an affine transformation. A fourth reference implant non‐coplanar with the first three implants would allow a full three‐dimensional coordinate system. In addition, the perceived movements were substantially larger for the controls, likely due to them being farther away from the reference implants, making them a worst‐case control instead of a one‐to‐one comparison. The placement of additional reference implants around the control implants would help ensure that these measurements are improved upon. We wished to avoid placing too many implants in the animal to avoid issues with inflammation and due to limits in the available space within each target muscle, but additional reference implants may have been warranted had we not had the primary histological endpoint. Finally, and most importantly, it should be noted that the use of position reference markers within a muscle to track the movement of a marker within a muscle will be subject to muscle remodeling due to growth, exercise, or disuse [29]. This can be observed in the greater perceived movements in the first portion of the CT imaging comparison, which had a larger delay between the CT images (8 vs. 3 days) and is also supported by the observation that the animal's final body weight increased by 46% (from 54 to 79 kg) over the approximately 6‐week duration of the study, suggesting growth and remodeling were likely contributing factors. These factors might be partially corrected for by reducing the amount of time between CT scans. For instance, CT scans could be performed immediately before and immediately after MRI and then again 48 h post‐exposure. A future experimental design might also use high‐resolution magnet tracking to sense the strength and orientation of the permanent magnet implants pre and post exposure.

## 7 Conclusion

Under 1.5‐T MRI exposure, histological analysis suggested that the implanted magnets did not migrate through tissue, worst‐case demagnetization (rigidly affixed in an opposing orientation) was 13.5%, and maximum image artifacts observed were 71 mm. Direct applicability of these results is limited to 1.5‐T MRI or below and to the specific implants tested in muscle. The lack of migration, resistance to demagnetization, and size of image artifacts that we observed in this investigation suggest that these 3‐mm‐diameter, intramuscular, permanent‐magnet implants do not preclude a patient from being imaged by MRI at a strength at or below 1.5T, showing the potential for safe scanning and future MR Conditional labeling for these implants.

## Conflicts of Interest

C.R.T., S.H.Y., C.C.S., and H.M.H. have patents related to the implantation of permanent magnet implants in muscle. E.D.A., S.J.C., and D.C.G. are employed by MED Institute. A.F.T., J.O.N.‐G., C.E.S., L.T.L., and S.R.H. are employed by Cook Research Incorporated.

## Supporting information


**Figure S1:** Empirical testing of magnetically induced displacement force. (A) Diagram (B) Photograph.
**Figure S2:** Empirical testing of magnetically induced torque. (A) Diagram (B) Photograph.
**Figure S3:** Representative image of a test site. Post implant photograph illustrating the position of the sutures used to secure implants in the left semimembranosus muscle. The site of the permanent magnet implant (circled in gold) is surrounded by 3 position reference implants (circled in green). The nonmagnetic control implant was placed at the other end of the test site (circled in blue).
**Table S1:** Transformed position measurement changes in millimeters from pre to post exposure at 0.55T and 1.5T. While the results are grouped by muscle for convenience, note that this should not be construed to convey that there are any substantial one‐to‐one relationships between a permanent magnet and its neighboring same‐muscle control.
**Figure S4:** Empirical testing of image artifacts. (A) Diagram (B) Photograph.
**Figure S5:** Simulated effect of 1.5‐T‐induced partial demagnetization on distance tracking error between two magnets at various depths. The mean absolute distance errors were calculated by simulating the tracking of two magnets with state‐of‐the‐art hardware and software. At the clinically relevant depth of 45 mm, a partial demagnetization from 1.39 down to 1.20 nT/m^3^ resulted in an increase in the distance tracking error from approximately 2.07 mm up to 2.42 mm, an increase of approximately 17%.
**Figure S6:** Empirical versus simulation demagnetization results. Simulation was of a 3‐mm spherical permanent magnet with parameters *B*_r_ = 1393 mT, *H*_cb_ = 1093 kA/m, *H*_ci_ = 1637 kA/m, and (BH)_max_ = 381.38 kJ/m^3^.
