# Supplementary material for: MRI Safety Considerations for Permanent Magnet Implants in Muscle
Source: J Magn Reson Imaging. 2025 Sep 17;63(2):563–73. doi: 10.1002/jmri.70126 (PMC12810996; doi:10.1002/jmri.70126)
Supplement: Supplementary file 1 — Figure S1: Empirical testing of magnetically induced displacement force. (A) Diagram (B) Photograph. Figure S2: Empirical testing of magnetically induced torque. (A) Diagram (B) Photograph. Figure S3: Representative image of a test site. Post implant photograph illustrating the position of the sutures used to secure implants in the left semimembranosus muscle. The site of the permanent magnet implant (circled in gold) is surrounded by 3 position reference implants (circled in green). The nonmagnetic control implant was placed at the other end of the test site (circled in blue). Table S1: Transformed position measurement changes in millimeters from pre to post exposure at 0.55T and 1.5T. While the results are grouped by muscle for convenience, note that this should not be construed to convey that there are any substantial one‐to‐one relationships between a permanent magnet and its neighboring same‐muscle control. Figure S4: Empirical testing of image artifacts. (A) Diagram (B) Photograph. Figure S5: Simulated effect of 1.5‐T‐induced partial demagnetization on distance tracking error between two magnets at various depths. The mean absolute distance errors were calculated by simulating the tracking of two magnets with state‐of‐the‐art hardware and software. At the clinically relevant depth of 45 mm, a partial demagnetization from 1.39 down to 1.20 nT/m3 resulted in an increase in the distance tracking error from approximately 2.07 mm up to 2.42 mm, an increase of approximately 17%. Figure S6: Empirical versus simulation demagnetization results. Simulation was of a 3‐mm spherical permanent magnet with parameters B r = 1393 mT, H cb = 1093 kA/m, H ci = 1637 kA/m, and (BH)max = 381.38 kJ/m3. [file JMRI-63-563-s001.docx]

**Supplementary Materials**

**Supplementary Section 1**

We performed Magnetically Induced Displacement Force testing following ASTM F2052-21 (see Supplementary Figure 1). We placed the magnet inside a ballast and used a string to suspend the ballast from a protractor. We used a four-bar linkage to maintain the protractor level while positioning the magnet along the center axis of the MRI system at a measured z-position outside the bore. We then measured the angle of the string, 𝛼, with respect to vertical and calculated the magnetically induced force, *F*, as

*F* = (*m*_magnet_ + *m*_ballast_) *g* tan(𝛼),

where *m*_magnet_ and *m*_ballast_ are the masses of the magnet and ballast, respectively, and *g* is the acceleration on earth due to gravity.

We measured a magnetic field magnitude and gradient of 0.43 T and 0.80 T/m at the location of 0.55-T imaging exposure, and a magnetic field magnitude and gradient of 1.04 T and 2.80 T/m at the location of the 1.5-T imaging exposure. To calculate the magnetically induced force at 0.55 T and 20.00 T/m for the 0.55-T test and to 1.50 T and 20.00 T/m for the 1.5-T test, we first converted the angle of the string to these desired exposure parameters as

𝛼_desired_ = tan^-1^ ( tan(𝛼_measured_) B_desired_ (dB/dz)_desired_ / (B_measured_ (dB/dz)_measured_) )

We repeated each measurement three times and logged the median as the result.


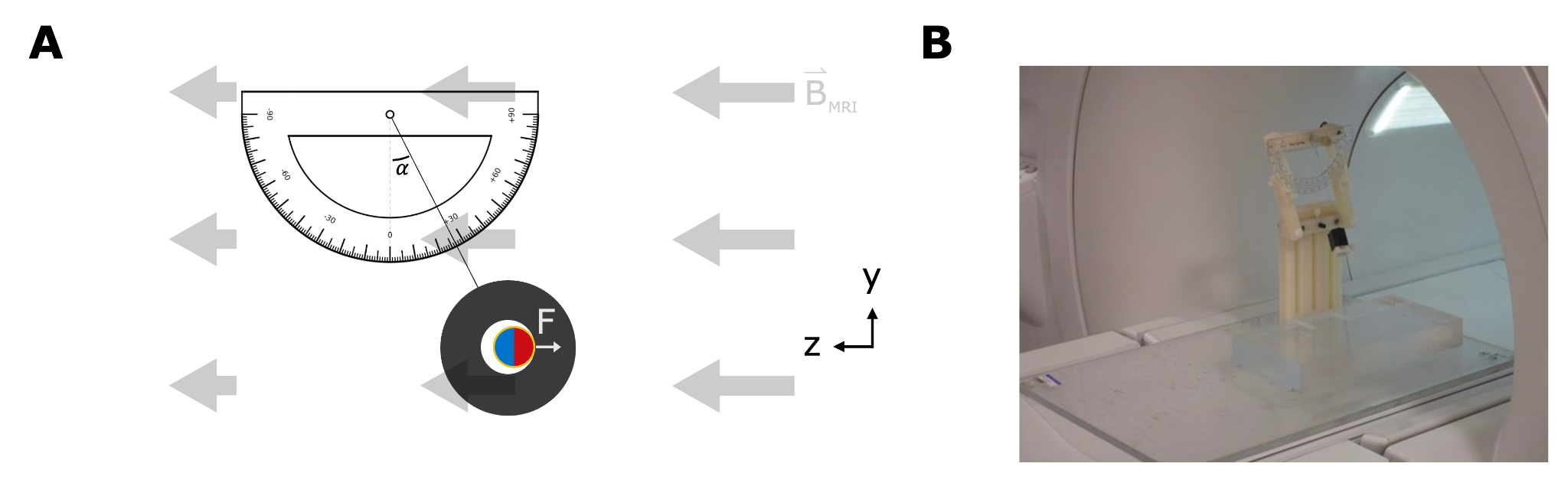


**Supplementary Figure 1: Empirical Testing of Magnetically Induced Displacement Force.** (A) Diagram (B) Photograph

We performed Magnetically Induced Torque testing following ASTM F2213-17 (see Supplementary Figure 1). We affixed the magnet to a rotating plate with a pulley string attached at a distance *r* = 0.0262 m from its center of rotation, then positioned the plate and pulley within the MRI so that the magnet was located at isocenter. Finally, we slowly rotated the plate by pulling on the string with a force gauge, calculated the maximum magnetically induced torque, 𝜏, as

𝜏 = *r* (*F*_pulley_ - *F*_friction_),

where *F*_pulley_ was the maximum force measured with the magnet present, and *F*_friction_ was the force due to friction measured without the magnet present. We repeated each measurement three times and logged the median as the result.


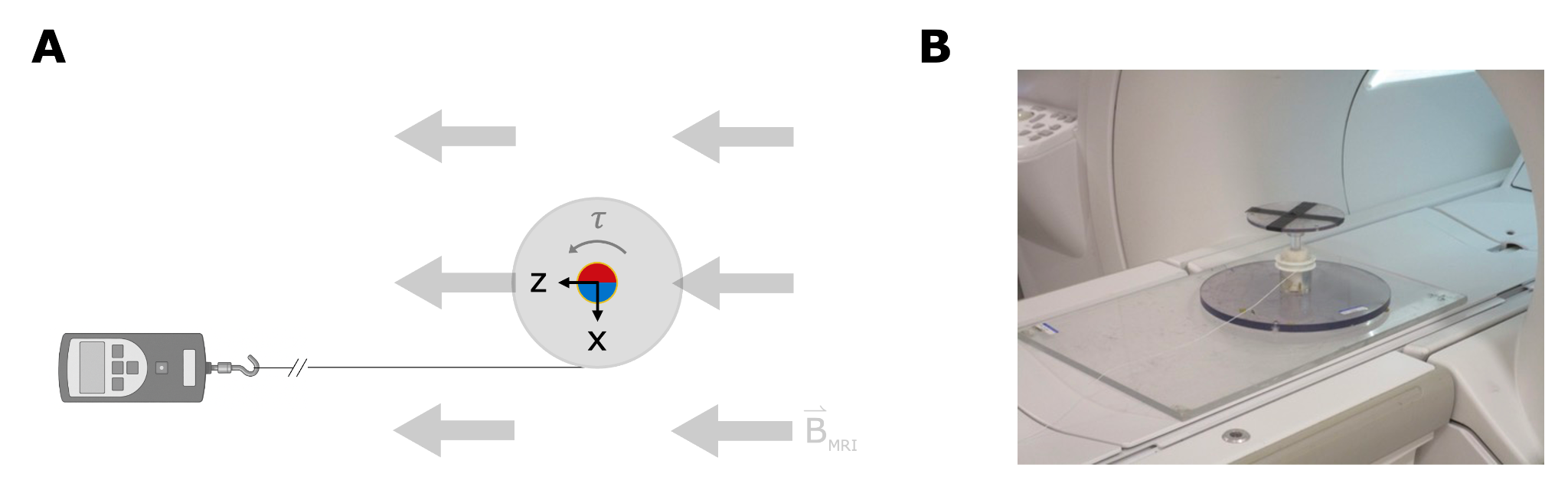


**Supplementary Figure 2: Empirical Testing of Magnetically Induced Torque.** (A) Diagram (B) Photograph


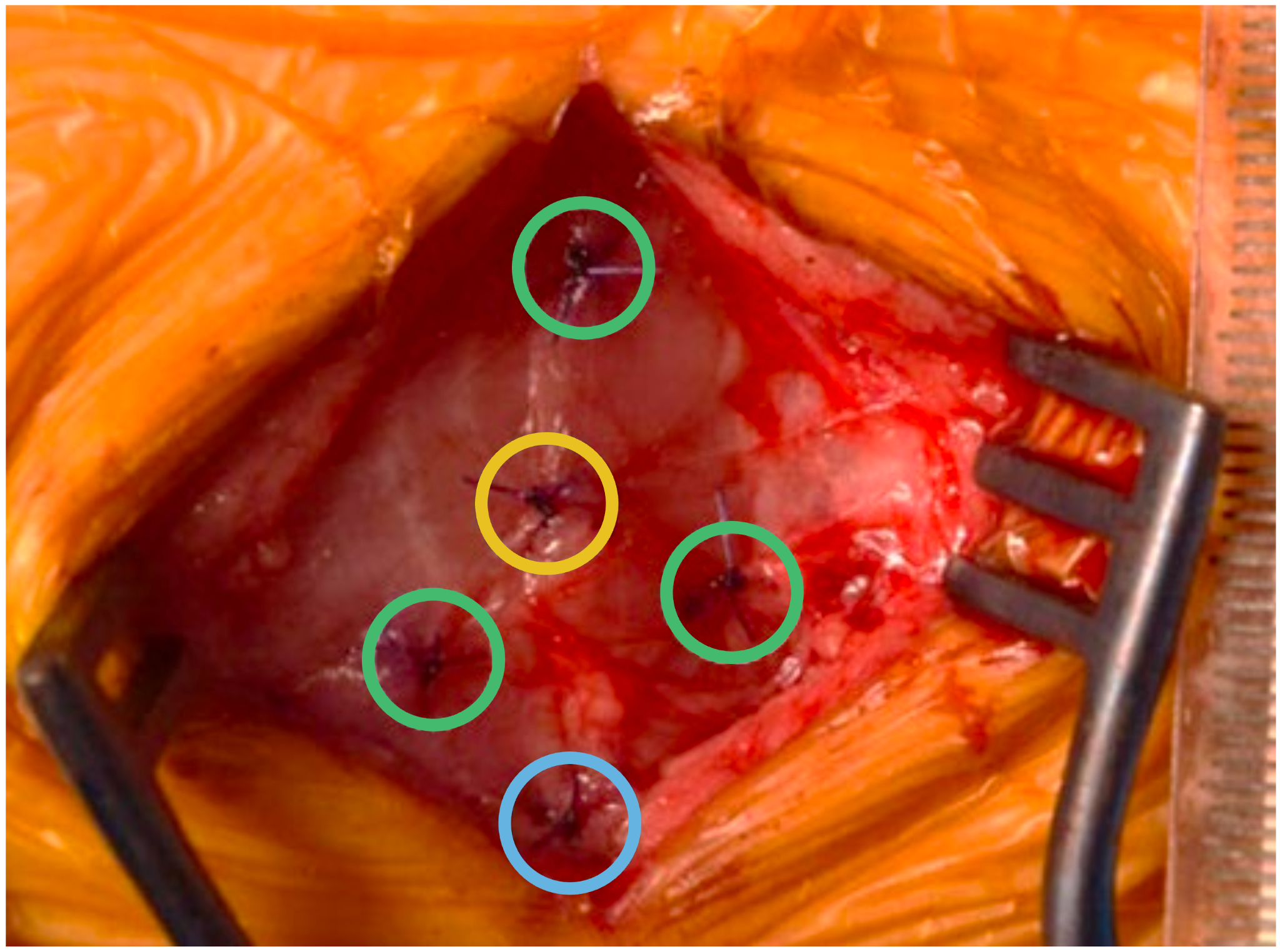


**Supplementary Figure 3: Representative image of a test site.** Post implant photograph illustrating the position of the sutures used to secure implants in the left semimembranosus muscle. The site of the permanent magnet implant (circled in gold) is surrounded by 3 position reference implants (circled in green). The nonmagnetic control implant was placed at the other end of the test site (circled in blue).

**Supplementary Section 2**

The animal was in a prone position for all CT images. In an attempt to account for differences in animal placement in the CT imaging, during each CT imaging session, we repeatedly fully removed the animal from the CT table, returned it to the CT table in its imaging position, and reimaged it. We repeated this process during each imaging session until five CT scans had been taken of the animal. We transported and imaged the animal under anesthesia for all exposure and imaging. We used a slice thickness of 0.625 mm for all CT images.

To compare measured implant positions from before to after MRI exposure, we imported all five CT images before exposure and all five CT images after exposure using PyDicom. We performed the following analysis separately for the 0.55-T case and the 1.5-T case. We applied an initial thresholding to the images at 90% of the relative pixel intensity and used connected components labeling to detect all 40 magnetic, control, and reference implants (five implants in each of eight muscles), and used distance filtering to merge implants that were detected more than once. We then smoothed the already-thresholded data with a three-dimensional gaussian filter (𝜎 = 2 pixels), locally re-thresholded each implant to 80% of its neighboring relative pixel intensities, and calculated the volume and center location of each implant. We then used *k*-means clustering to group the implants according to muscle (into eight groups of five) and categorized the implants as magnetic, control, or reference based on their calculated volumes and relative positions within each group. For each muscle, the magnetic and control implant positions from all scans were then transformed into the coordinate system of the first of the five before-scans via an affine transformation guided by the position reference implants. For each magnetic and control implant, its before and after x, y, and z coordinates were selected independently as the median of its five coordinate measurements from the five before or after scans, respectively. Finally, a transformed position measurement change was calculated as the cartesian distance between the median before-position and median after-position of each implant.

The unsorted Euclidean distances arranged according to exposure level test and muscle grouping can be seen in Supplementary Table 1.

**Supplementary Table 1. Transformed position measurement changes in millimeters from pre to post exposure at 0.55 T and 1.5 T.** While the results are grouped by muscle for convenience, note that this should not be construed to convey that there are any substantial one-to-one relationships between a permanent magnet and its neighboring same-muscle control.


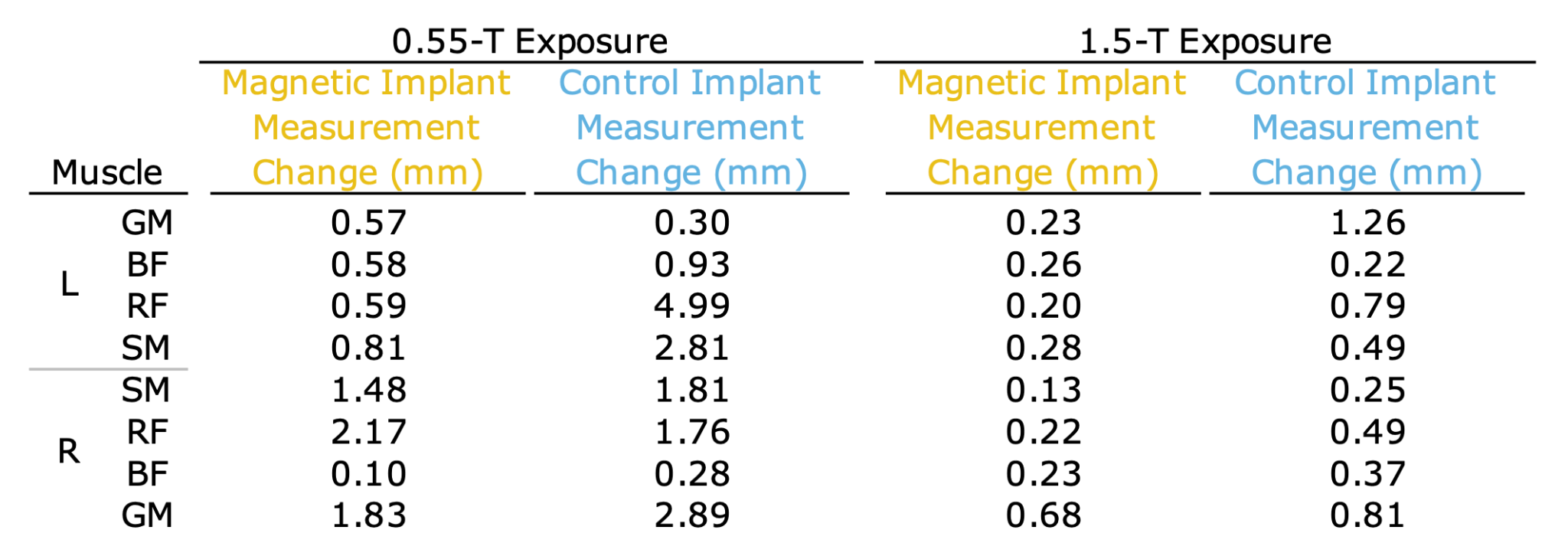


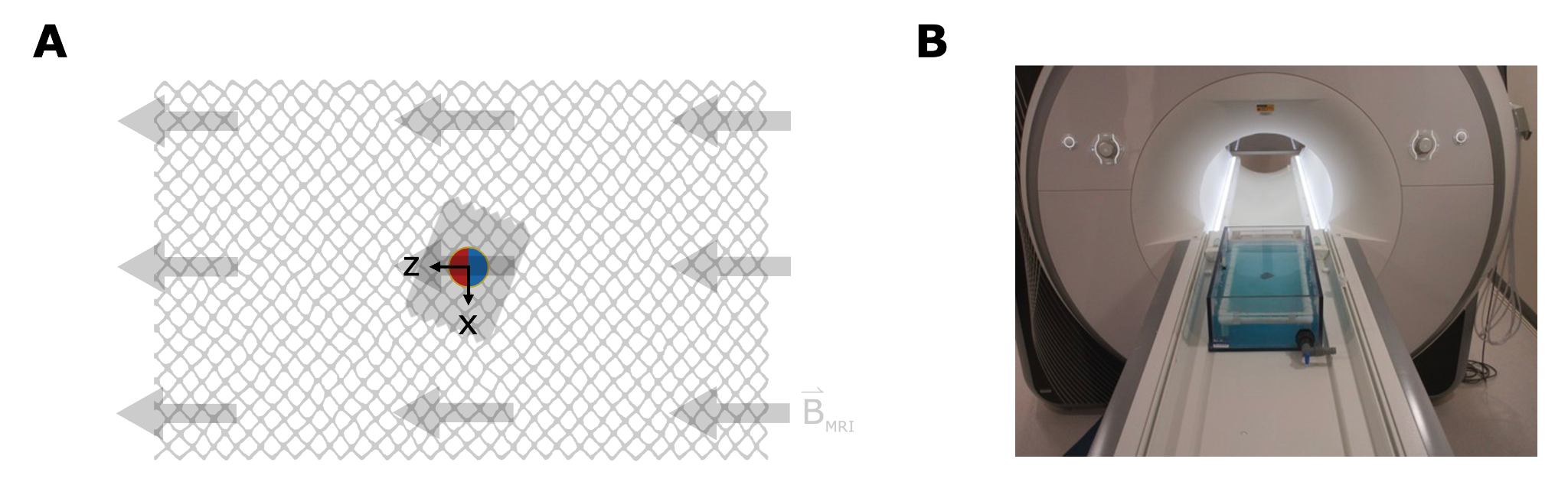


**Supplementary Figure 4. Empirical Testing of Image Artifacts.** (A) Diagram (B) Photograph

**Supplementary Section 3**

*Gross Findings*

Examined sections of the left and right biceps femoris, left rectus femoris, left and right middle gluteal, and left and right semimembranosus muscles were within normal limits. An approximately 6.5 cm diameter seroma was identified within the superficial fascia of the right rectus femoris; this muscle contained a magnetic implant and a control implant. On cut section, approximately 100 mL of clear fluid exuded from the seroma.

*Nonmagnetic Control Implant Histopathology*

The control implant sites were surrounded by a thin fibrous capsule that measured between 20−220 µm in thickness. The fibrous capsule was expanded by small numbers of lymphocytes, plasma cells, and occasionally macrophages. In some areas the fibroblasts extended beyond the implant site and entrapped some myocytes. A small number of myocytes had a regenerative profile and were multinucleated. The control implant coating was visible in all sections with minimal biological response. The surrounding skeletal muscle was normal.

In the right rectus femoris muscle, which was associated with the grossly described seroma, there was a 3.5 × 2.2 mm irregularly shaped segment of granulation tissue distant from the control implant site. The granulation tissue was composed of fibroblasts and infiltrated by small numbers of lymphocytes and plasma cells. Few myocytes were entrapped within the fibroblasts. Some of these entrapped myocytes showed evidence of regeneration with a multinuclear profile.

Overall, the control implant sites showed evidence of advanced healing and were biocompatible with an appropriate biological response. There was no histologic evidence of migration of the Parylene-C-coated tantalum control implants. Skeletal muscle necrosis was absent in all sections.

*Permanent Magnet Implant Histopathology*

The permanent magnet implant sites were spherical and lined circumferentially by a thin fibrous capsule that measured between 20−500 µm thick. The fibrous capsule was mildly infiltrated by lymphocytes, plasma cells, and occasionally multinucleated giant cells. Some myocytes were entrapped within the fibrovascular capsule. Some of the entrapped myocytes had a regenerative, multinucleated profile. Magnetic implant coating was visible in all sections with minimal biological response. Myonecrosis was absent.

In the right rectus femoris, there was a 3.2 mm thick segment of granulation tissue, adjacent to the grossly described seroma that occupied the length of the examined muscle section. The granulation tissue included fibroblasts, clusters of lymphocytes and plasma cells, and small numbers of multinucleated giant cells. Thin, fingerlike projections of fibroblasts connected the granulation tissue to the permanent magnet implant site.

Overall, the permanent magnet implant sites showed evidence of advanced healing. The magnetic implants were biocompatible with an appropriate biological response. There was no histologic evidence of migration of the magnetic implants. Skeletal muscle necrosis was absent in all sections.

**Supplementary Section 4**

We simulated the effect of the maximum observed empirical partial demagnetization induced by 1.5-T exposure. Specifically, we simulated the tracking of the distance between two magnets, where both magnets are first tracked with their full magnetization, and then tracked with a magnetization reduced by 13.1% (see Supplementary Figure 5). The distance tracking error resulting from the magnetization reduction was 17%.

**
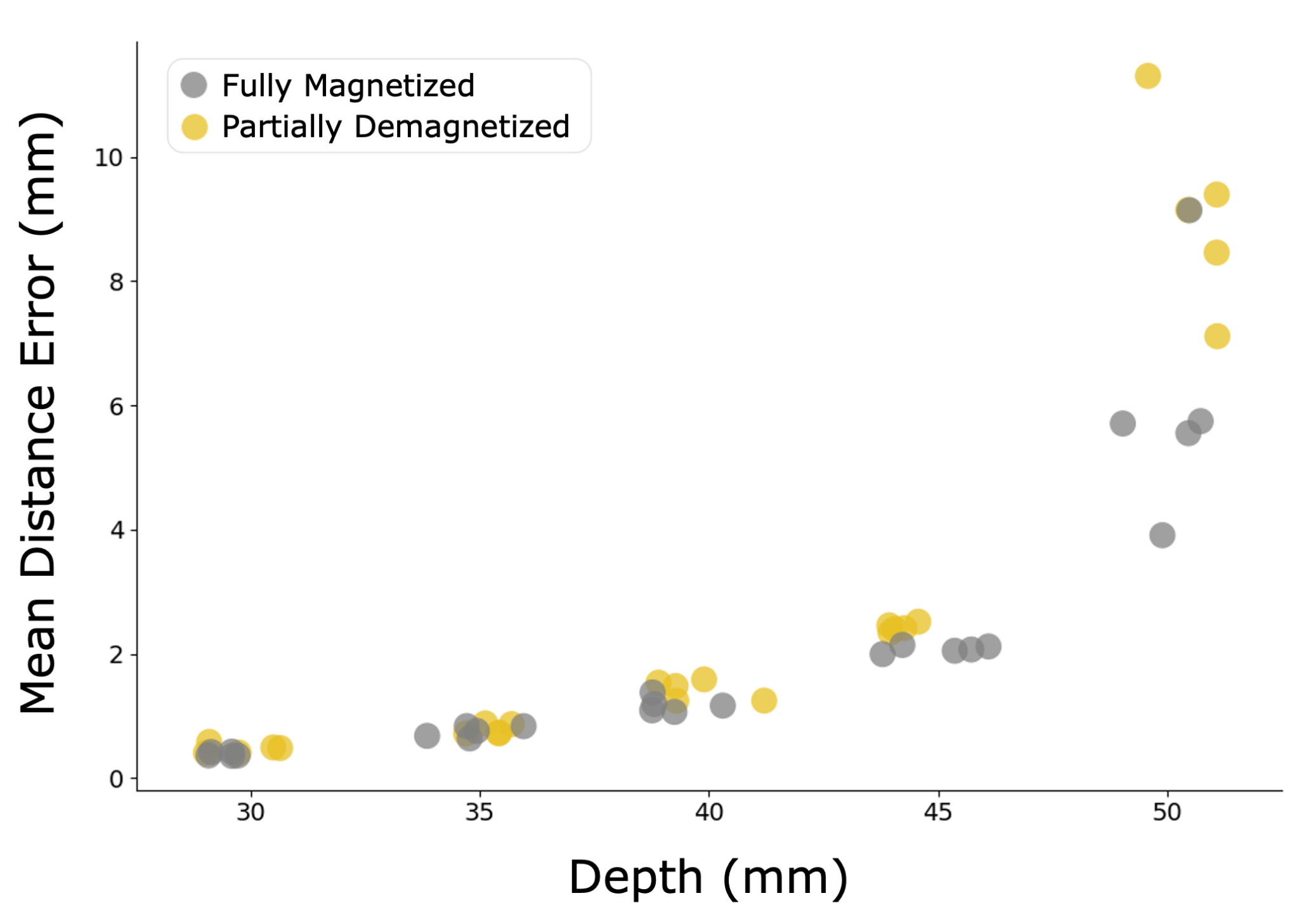
**

**Supplementary Figure 5:** Simulated effect of 1.5-T-induced partial demagnetization on distance tracking error between two magnets at various depths. The mean absolute distance errors were calculated by simulating the tracking of two magnets with state-of-the-art hardware and software. At the clinically relevant depth of 45 mm, a partial demagnetization from 1.39 nT/m^3^ down to 1.20 nT/m^3^ resulted in an increase in the distance tracking error from approximately 2.07 mm up to 2.42 mm, an increase of approximately 17%.

**Supplementary Section 5**

We wish to derive the magnitude of an applied magnetic flux density, *B*_applied_ = *μ*_0_ *H*_applied_, that would demagnetize the implanted permanent magnets. To account for the geometry and magnetization of the permanent magnet, we note that

*H*_effective_ *= H*_applied_ *- N M*,

where the magnetization *M* is given by *M = B*_r_ */ μ*_0_, and where *N* is the demagnetization factor. To demagnetize the magnet, the effective magnetic field must be opposite the magnetization and exceed the coercivity, as *H*_effective_ *= -H*_ci_.

Substituting in these relationships, solving for *B*_applied_, and substituting in specific values for the spherical magnets of this study (*B*_r_ = 1.393 T and *H*_ci_ = 1.637 MA/m, N = 1/3) gives

*-H*_ci_ *= H*_applied_ *- N (B*_r_ */ μ*_0_*)*

*H*_applied_ *= N (B*_r_ */ μ*_0_*) - H*_ci_

*B*_applied_ *= N B*_r_ *- μ*_0_ *H*_ci_

*B*_applied_ *=* 0.464 - 2.06 T

*B*_applied_ *= -*1.59 T

This suggests that permanent magnets with these magnetic properties and geometry may not be substantially demagnetized by exposure to 0.55-T or 1.5-T MR imaging under worst-case conditions, but could be substantially demagnetized by exposure to imaging at higher magnetic flux density strengths (e.g., 3-T or 7-T MR imaging).

Based on this preliminary analysis, we performed a preliminary simulation and data collection to verify that under worst-case conditions (permanent magnets fixed at some given angle), it was possible to fully demagnetize the magnets under exposure to 3-T magnetic fields (see Supplementary Figure 6).

**
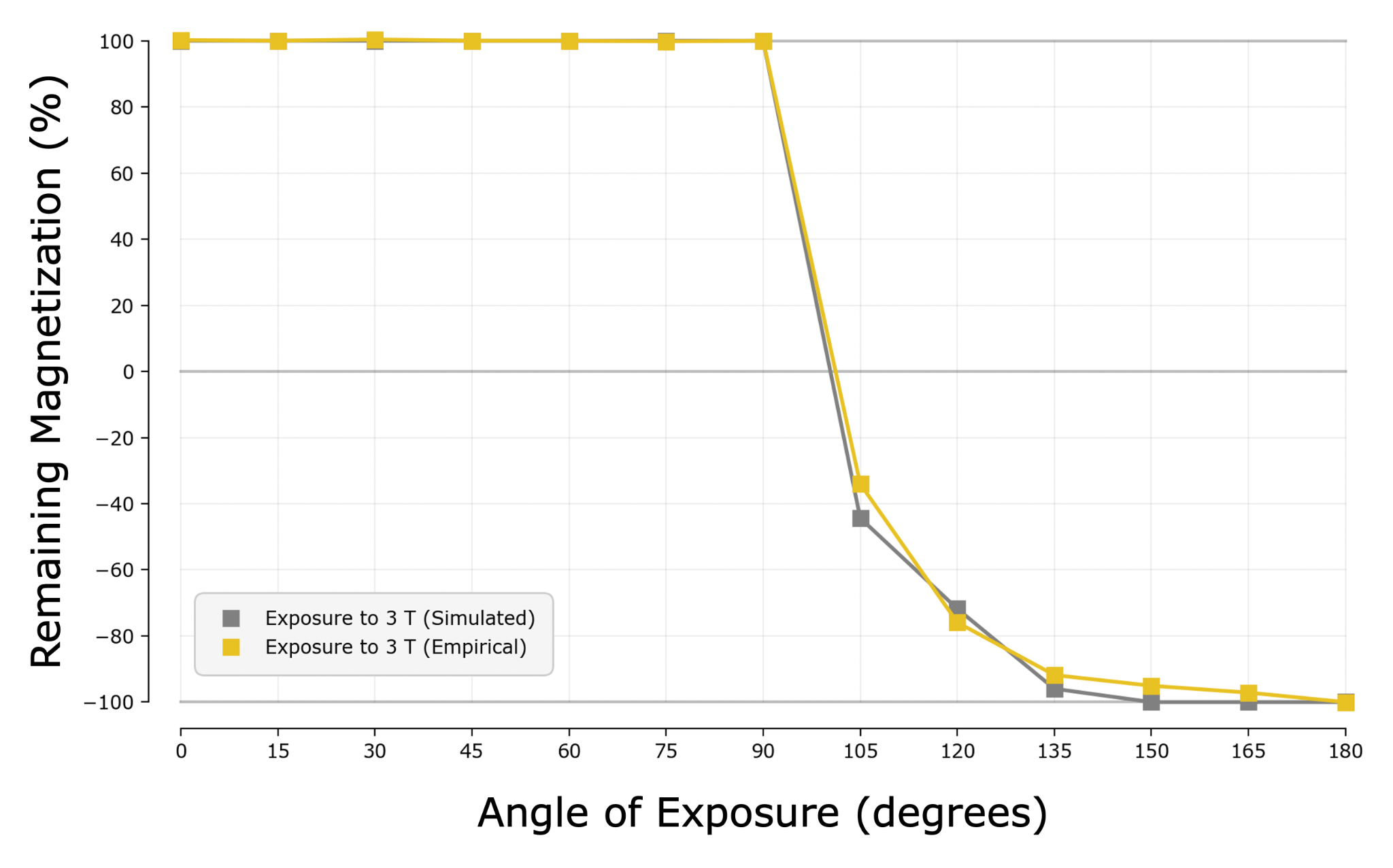
**

**Supplementary Figure 6.** Empirical versus simulation demagnetization results. Simulation was of a 3-mm spherical permanent magnet with parameters B_r_ = 1393 mT, H_cb_ = 1093 kA/m, H_ci_ = 1637 kA/m, and (BH)_max_ = 381.38 kJ/m^3^.

The results of our preliminary study exposing the magnetic implants to 3-T magnetic flux density revealed that it is possible to fully demagnetize the magnets under worst case conditions (fixed at roughly 100 degrees relative to the applied field [oriented slightly opposite the field]). Based on these results, we decided to test 1.5-T exposure in this work experiment and leave further investigation of 3-T exposure to future work.
